# Supplementary material for: Systematic characterization of short intronic splicing-regulatory elements in SMN2 pre-mRNA
Source: Nucleic Acids Res. 2022 Jan 8;50(2):731–49. doi: 10.1093/nar/gkab1280 (PMC8789036; doi:10.1093/nar/gkab1280)
Supplement: gkab1280_Supplemental_Files [file gkab1280_supplemental_files.zip › Supplementary Figures and Table S3-S9.pdf]

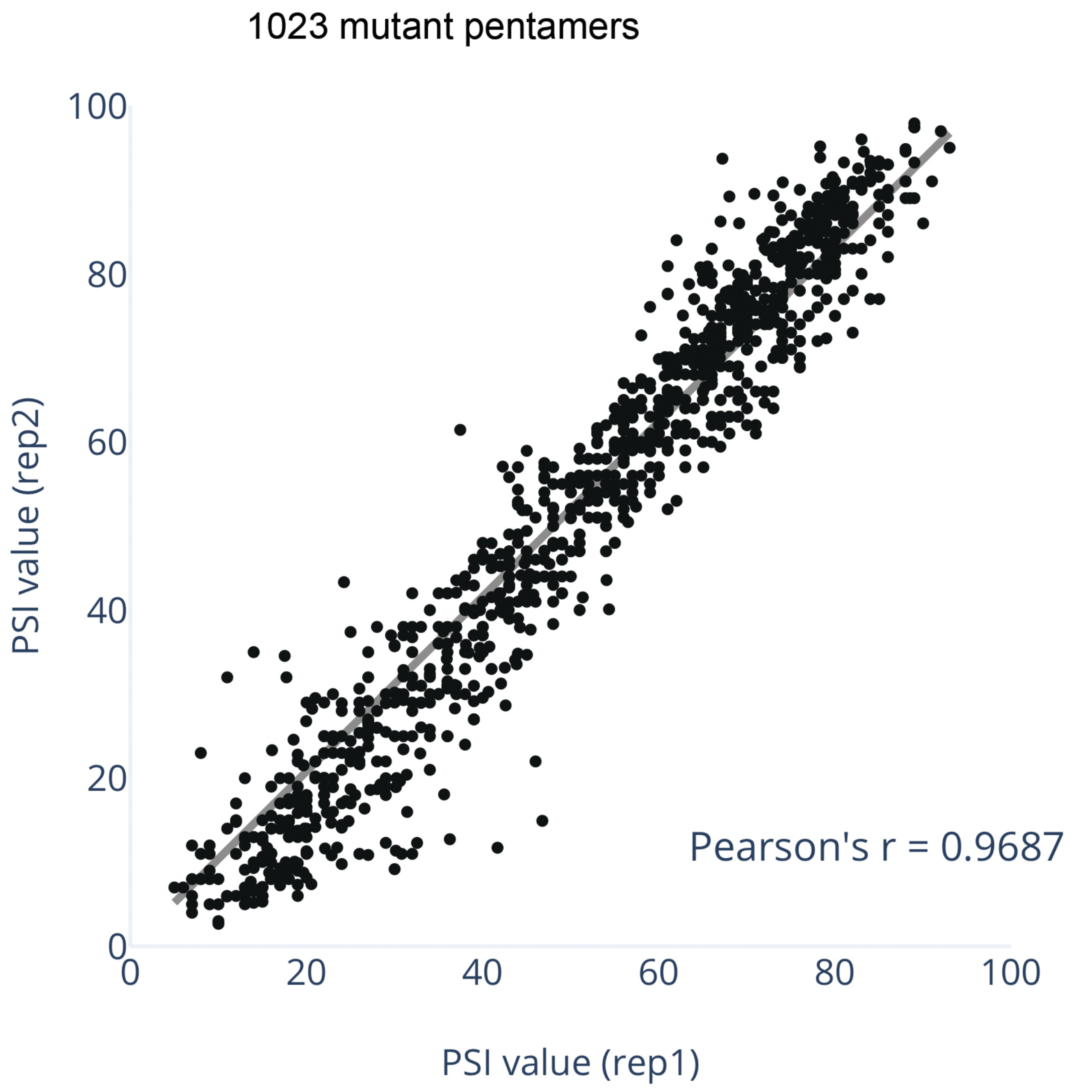

Figure S1. Scatterplot of PSI values of 1,023 mutant pentamers in two replicates. Each block dot represents on mutant pentamer. X- and Y-axes show the PSI value of *SMN2* exon 7 inclusion in two replicates (rep1 and rep2), respectively. Linear regression was used to calculate the R^2 value while intercept was set to zero (y = 1.0405x).

Supplementary Figure S2

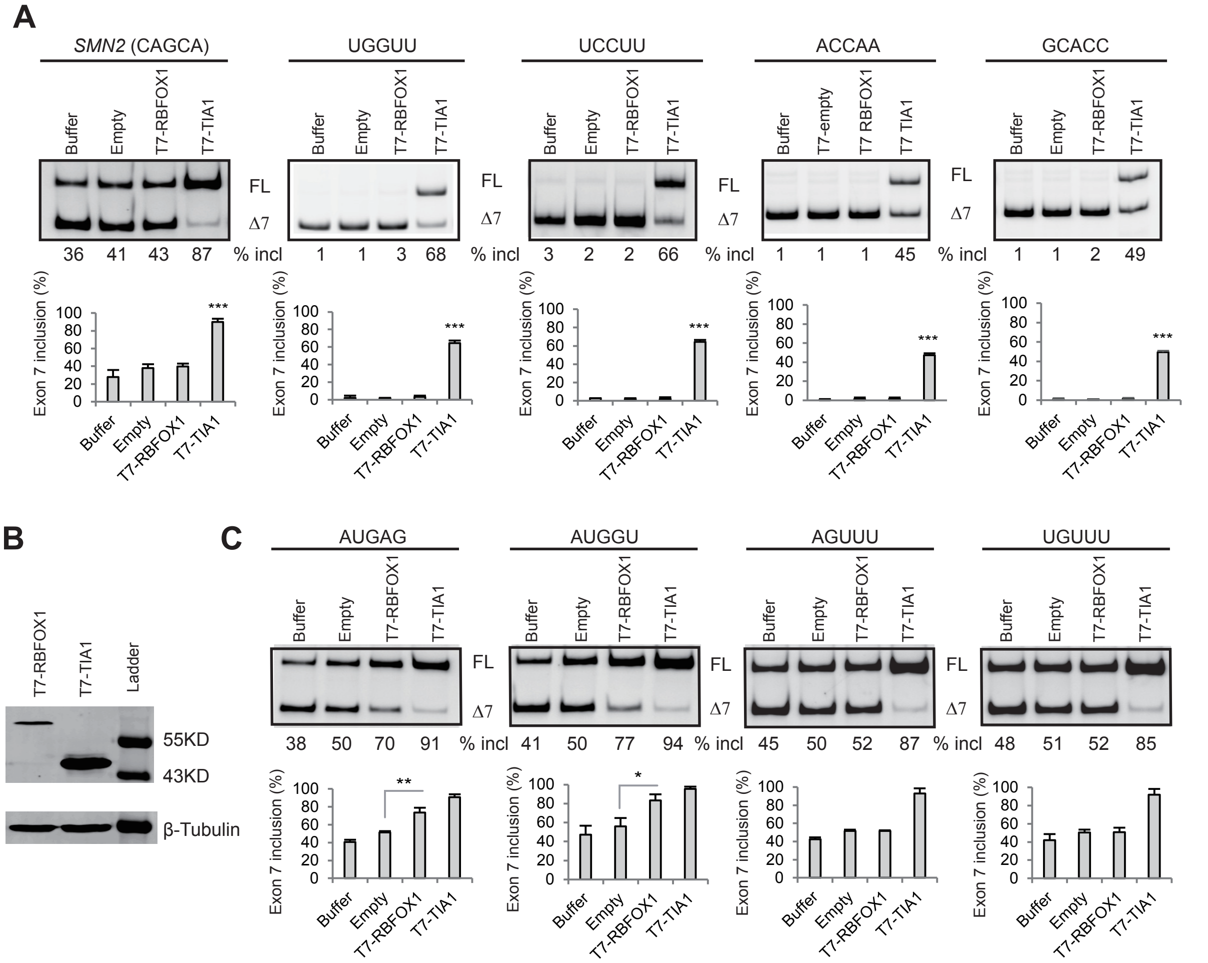

Figure S2. Effects of TIA1 and Rbfox1 on exon 7 splicing of *SMN2* minigene mutants with the mutated pentamers being U-rich or the first trinucleotide in pentamers being AUG. Each mutant was co-transfected with a protein-expression plasmid into HEK293 cells and exon 7 splicing was analyzed by Cy5-labeled RT-PCR. **A**. Two pentamers containing 3 Us were compared with two without a U. The exon 7 inclusion percentages for all the four mutants were similar and very low due to a potential RNA structure or inhibitory motifs. T7-TIA1 overexpression resulted in better exon 7 splicing when more Us were present in these pentamers, suggesting TIA1 competing with splicing repressors or other inhibitory contributors to bind to the U-rich sequences and promote splicing. Note, TIA1 overexpression promoted exon 7 splicing of the WT *SMN2* minigene because of the presensce of U-rich motifs downstream of the library-building site in intron 7 (Singh NN, et al. 2011 Mol Cell Biol). **B**. T7-tagged proteins were properly expressed. **C**. T7-Rbfox1 promoted exon 7 splicing for only mutants with AUG as the first trinucleotide. Note, the upstream trinucleotide is UGC and they form UGCAUG at the junction. Empty: empty vector, FL: full length, Δ7: exon 7-skipped isoform, % incl: percentage of exon 7 inclusion. *** $P < 0.001$, ** $P < 0.01$, * $P < 0.05$, compared to empty vector (pCGT7) control.

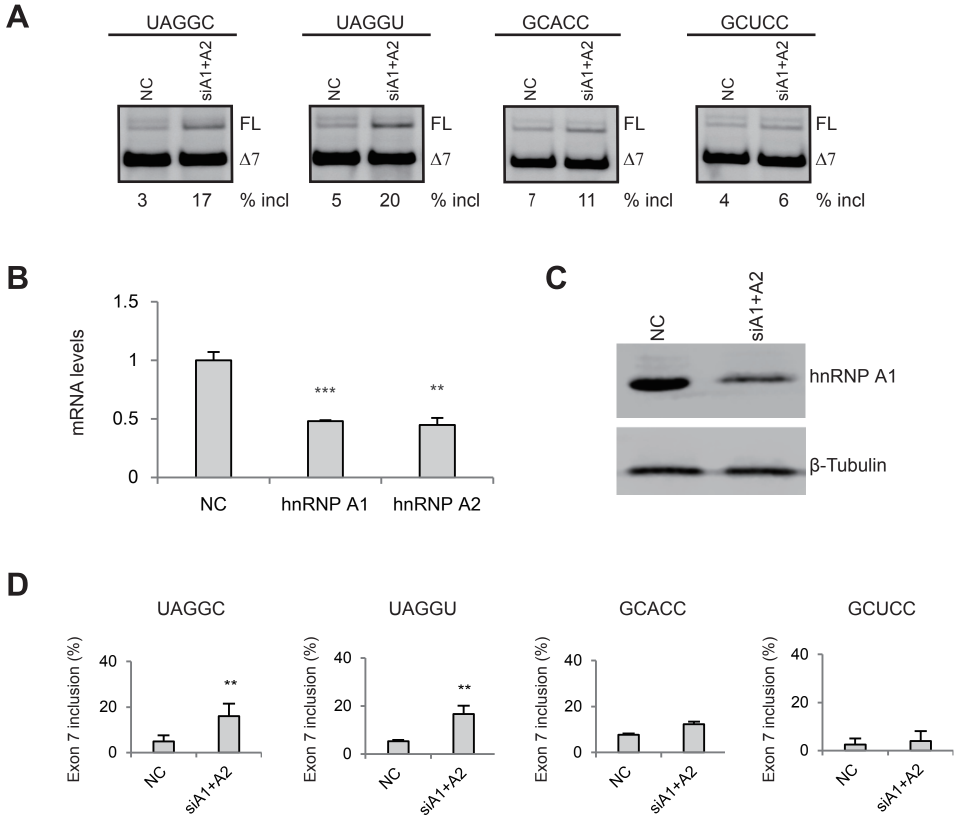

Figure S3. Knockdown of hnRNP A1 and A2 levels promoted more exon 7 inclusion in *SMN2* minigene mutants with the mutated sequences containing hnRNP A1 motifs (such as UAGG) than mutants with the mutated sequences containing none of hnRNP A1 motifs. **A**. Each of the four *SMN2* minigene mutants was co-transfected with two previously reported siRNAs against hnRNP A1 (siA1) and A2 (siA2), respectively (Cartegni et al. 2006 Am J Hum Genet), in HEK293 cells. A non-related siRNA (Sense: 5′-UUCUCCGAACGUGUCACGUTT-3′) was used as a control. Exon 7 splicing was analyzed with Cy5-labelled RT-PCR. **B.** hnRNP A1 and A2 mRNA levels were analyzed by quantitative RT-PCR after siRNA treatment. Primers for hnRNP A1: A1-F (5′-ACAACTTCGGTCGTGGAGGAAACT-3′) and A1-R (5′-CCAAAATTGCTTCC ATCATTACCAA-3′); primers for hnRNP A2: A2-F (5′-GCTGTAGCAAGAGAGGAATC TGGA-3′ and A2-R (5′-GCTTCTTCACAGTTACATGAGCCC-3′). **C**. Western blot detected robust reduction in hnRNP A1 protein levels after siRNA treatment. **D**. Quantitation of the data in panal A. FL: full length; Δ7: exon 7-skipped isoform; % incl: percentage of exon 7 inclusion. ** $P < 0.01$, *** $P < 0.001$.

Supplementary Figure S4

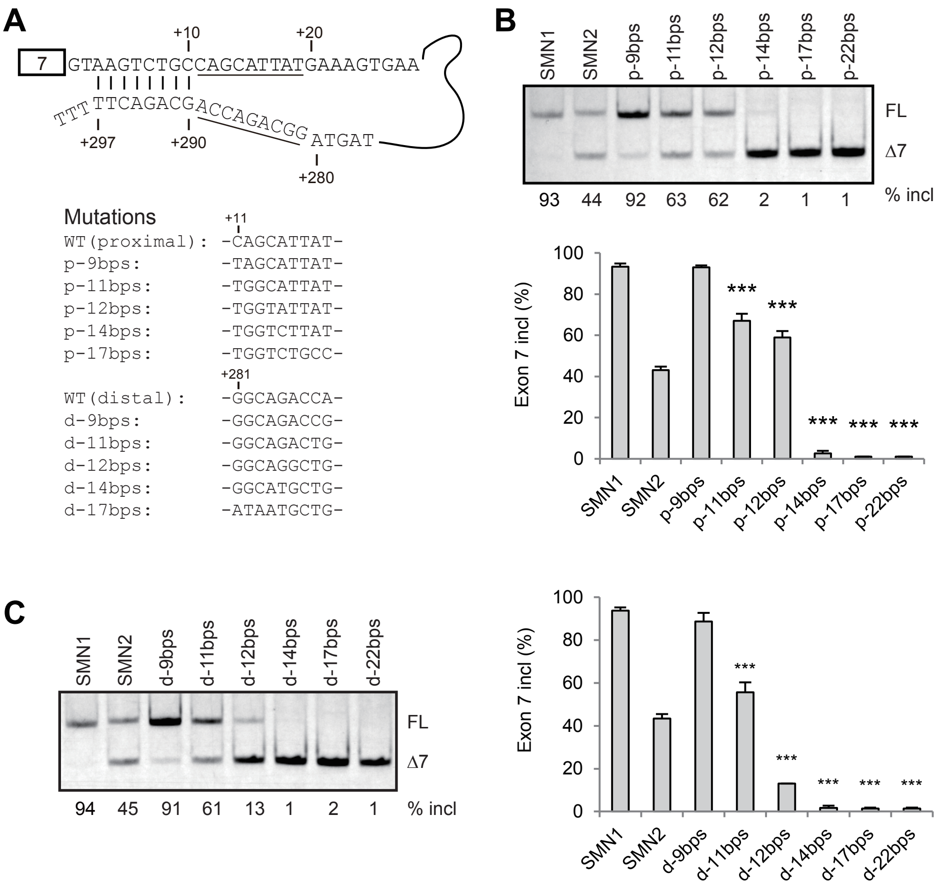

Figure S4. Strengthening the putative dsRNA in *SMN1* intron 7 inhibits exon 7 splicing. **A**. *SMN1* minigene mutants and their sequences were shown. Analysis of exon 7 splicing for mutations in the proximal (**B**) and distal (**C**) strands of the dsRNA structure in HEK293 cells by Cy5-labeled RT-PCR. Histagrams clearly show an inverse correlation between the length of the dsRNA stretch and the percentage of exon 7 inclusion (% incl). FL: full length, Δ7: exon 7-skipped isoform. *** $P < 0.001$ compared to the WT *SMN2*.

Supplementary Figure S5

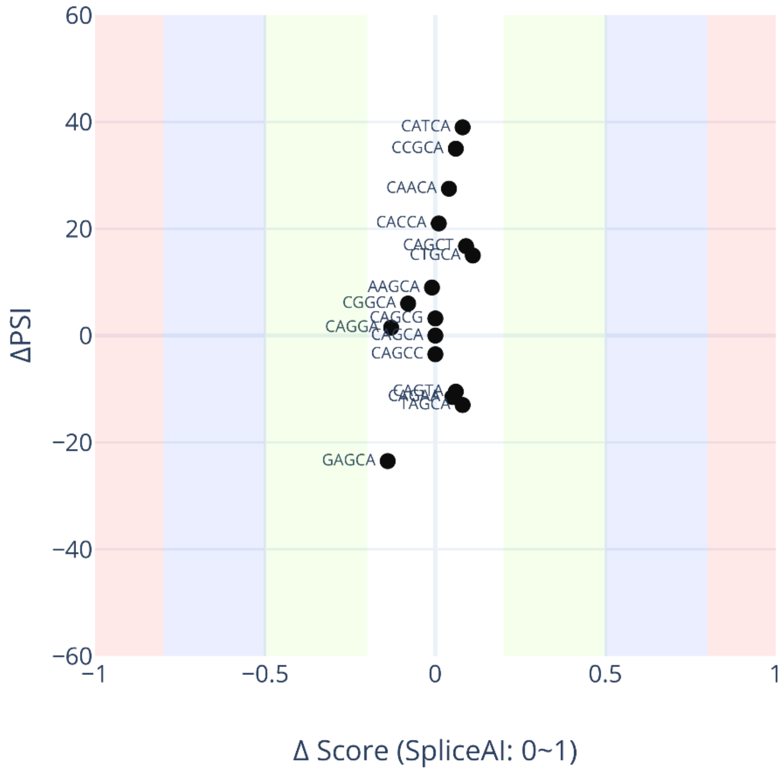

Figure S5. SpliceAI predictions (ΔScore) versus our minigene experiments (ΔPSI). 15 pentamers with single-nucleotide variations and the wild-type pentamer (CAGCA) *SMN2* minigene are shown here. Y-axis shows the ΔPSI values obtained from our minigene experiments, while X-axis shows the ΔScores based on SpliceAI's prediction. ΔScores of "Donor Gain" ranges from 0~1, and ΔScores of "Donor Loss" is transformed to 0~-1 to indicate exon skipping. Three color regions (red, blue, and green) show the range of confidence levels of SpliceAI cutoffs (0.2: high recall, 0.5: recommended, and 0.8: high precision). SpliceAI program was downloaded from GitHub (https://github.com/Illumina/SpliceAI). 15 pentamers with single-nucleotide variations and the wild-type minigene were used to create a .vcf file as input for SpliceAI predictions.

Supplementary Table S3. All 40 pentamers comprised of U and/or A and their ΔPSI values. Note, pentamers with more Us usually had much higher ΔPSI values.

Pentamer	ΔPSI	Pentamer	ΔPSI	Pentamer	ΔPSI	Pentamer	ΔPSI
AAAAA	10.50	AUAAA	16.50	UAAAA	1.50	UUAAA	10.50
AAAAU	27.00	AUAAU	39.50	UAAAU	27.00	UUAAU	31.50
AAAUA	22.00	AUAUA	33.50	UAAUA	25.50	UUAUA	41.50
AAAUU	35.50	AUAUU	44.50	UAAUU	37.50	UUAUU	46.00
AAUAA	14.00	AUUAA	28.50	UAUAA	11.00	UUUAA	23.50
AAUAU	42.00	AUUAU	49.00	UAUAU	36.00	UUUAU	50.50
AAUUA	33.00	AUUUA	51.00	UAUUA	37.00	UUUUA	57.00
AAUUU	45.50	AUUUU	51.50	UAUUU	47.50	UUUUU	58.50

Supplementary Table S4. All 40 pentamers comprised of U and/or C and their ΔPSI values. While most of these pentamers strongly promoted *SMN2* exon 7 splicing, some others were very inhibitory.

Pentamer	ΔPSI	Pentamer	ΔPSI	Pentamer	ΔPSI	Pentamer	ΔPSI
CCCCC	41.50	CUCCC	8.50	UCCCC	12.00	UUCCC	25.50
CCCCU	46.00	CUCCU	-3.50	UCCCU	24.02	UUCCU	38.05
CCCUC	46.00	CUCUC	41.00	UCCUC	-35.91	UUCUC	40.50
CCCUU	48.50	CUCUU	48.00	UCCUU	-24.50	UUCUU	58.50
CCUCC	-28.50	CUUCC	-30.50	UCUCC	-3.50	UUUCC	-12.50
CCUCU	39.23	CUUCU	21.78	UCUCU	38.97	UUUCU	-30.34
CCUUC	-11.50	CUUUC	31.39	UCUUC	42.27	UUUUC	45.48
CCUUU	44.00	CUUUU	52.50	UCUUU	55.50	UUUUU	58.50

Supplementary Table S5. List of all 16 pentamers containing UGGY motifs and their ΔPSI values.

Pentamer	ΔPSI	Pentamer	ΔPSI
UGGUA	-28.50	UGGCA	-26.00
UGGUC	-42.66	UGGCC	-31.00
UGGUG	-32.50	UGGCG	-26.47
UGGUU	-24.50	UGGCU	-27.48
AUGGU*	14.50	AUGGC*	-2.35
CUGGU	-20.00	CUGGC	-18.63
GUGGU	-15.00	GUGGC	-20.40
UUGGU	-19.00	UUGGC	-14.80

* They form RBFOX-binding motifs (UGCAUG) with the upstream trinucleotide UGC.

Supplementary Table S6. List of all 16 NUGGN pentamers (N = A, C, G or U) and their ΔPSI values.

Pentamer	ΔPSI	Pentamer	ΔPSI	Pentamer	ΔPSI	Pentamer	ΔPSI
AUGGA	39.50	CUGGA	-10.50	GUGGA	19.50	UUGGA	0.00
AUGGC	-2.35	CUGGC	-18.63	GUGGC	-20.40	UUGGC	-14.80
AUGGG	32.41	CUGGG	-4.84	GUGGG	34.01	UUGGG	0.61
AUGGU	14.5	CUGGU	-20.00	GUGGU	-15.00	UUGGU	-19.00

Supplementary Table S7. List of all 40 GGG-containing pentamers. 30 pentamers have a ΔPSI > 0; 18 have a ΔPSI > 17. Pentamers containing GGGG are shaded.

Pentamer	ΔPSI	Pentamer	ΔPSI	Pentamer	ΔPSI	Pentamer	ΔPSI
GGGAA	21.50	GGGGG	39.02	CGGGA	9.50	AUGGG	32.41
GGGAC	2.26	GGGGU	26.50	CGGGC	5.30	CAGGG	-23.89
GGGAG	1.48	GGGUA	30.50	CGGGG	27.98	CCGGG	27.95
GGGAU	16.00	GGGUC	4.17	CGGGU	18.50	CUGGG	-4.84
GGGCA	21.50	GGGUG	24.95	UGGGA	2.00	GAGGG	-13.96
GGGCC	5.50	GGGUU	4.50	UGGGC	-24.14	GCGGG	41.53
GGGCG	26.89	AGGGA	-33.00	UGGGG	1.34	GUGGG	34.10
GGGCU	20.34	AGGGC	-36.52	UGGGU	-21.50	UAGGG	-31.96
GGGGA	40.50	AGGGG	-17.97	AAGGG	3.94	UCGGG	1.10
GGGGC	27.23	AGGGU	-25.00	ACGGG	17.42	UUGGG	0.61

Supplementary Table S8. List of all 30 pentamers containing CGAC (CGACN and NCGAC) or creating CGAC with the upstream nucleotide C (GACNN) and their ΔPSI values.

Pentamer	ΔPSI	Pentamer	ΔPSI	Pentamer	ΔPSI	Pentamer	ΔPSI
GACAA	11.00	GACCG	39.75	GACUA	24.50	CGACG	24.44
GACAC	26.09	GACCU	37.62	GACUC	32.06	CGACU	14.11
GACAG	-16.15	GACGA	40.00	GACUG	32.36	ACGAC	4.41
GACAU	29.00	GACGC	40.36	GACUU	43.50	CCGAC	2.64
GACCA	26.00	GACGG	34.19	CGACA	13.00	GCGAC	-17.90
GACCC	17.50	GACGU	40.00	CGACC	7.50	UCGAC	-16.81

Supplementary Table S9. Analysis of all pentamers containing DGAC.

Pentamer	ΔPSI	Pentamer	ΔPSI	Pentamer	ΔPSI	Pentamer	ΔPSI
AGACA	-12.00	GGACG	-20.65	AAGAC	-28.06	GGGAC	2.26
AGACC	-16.50	GGACU	-20.00	CAGAC	-30.78	UGGAC	-36.34
AGACG	-14.15	UGACA	-5.50	GAGAC	-33.39	AUGAC	-23.24
AGACU	-12.78	UGACC	-1.50	UAGAC	-32.34	CUGAC	-21.67
GGACA	-18.00	UGACG	-17.09	AGGAC	-28.11	GUGAC	-33.02
GGACC	-6.00	UGACU	-9.01	CGGAC	16.24	UUGAC	-16.00
